# The Role of Bcl-2 Family Proteins and Sorafenib Resistance in Hepatocellular Carcinoma

**DOI:** 10.1155/2024/4972523

**Published:** 2024-08-19

**Authors:** Alex José de Melo Silva, Juliana Ellen de Melo Gama, Sheilla Andrade de Oliveira

**Affiliations:** Department of Immunology Aggeu Magalhães Institute, Recife, Pernambuco, Brazil

**Keywords:** Bcl-2 family, hepatocellular carcinoma, resistance, sorafenib

## Abstract

Liver cancer has been reported to be one of the most malignant diseases in the world. It is late diagnosis consequently leads to a difficult treatment, as the cancer reached an advanced stage. Hepatocellular carcinoma (HCC) is the primary type of cancer diagnosed in the liver, with deadly characteristics and a poor prognosis. The first-in-line treatment for advanced HCC is sorafenib. Sorafenib acts by inhibiting cell proliferation and by inducing apoptosis as well as blocks receptors associated with these mechanisms. Due to its constant use, sorafenib resistance has been described, especially to proteins of the Bcl-2 family, and their overexpression of Bcl-XL and Mcl-1. This review focuses on the role of the Bcl-2 proteins in relation to sorafenib resistance as a consequence of first-in-line treatment in HCC.

## 1. Introduction

Liver cancer is a malignant cancer and causes a serious global health problem, as being the sixth most common, and the third-ranked cause of death worldwide [1–4]. Hepatocellular carcinoma (HCC) is the primary cancer growing in the liver and the most prevalent form of all liver cancers [5]. HCC is diagnosed most of the time late and in an advanced stage, which consequently leads to a poor overall prognosis for the patient [6]. Furthermore, the disease develops in many patients from a chronically inflamed liver associated with various risk factors including viral infections, nonalcoholic steatohepatitis, alcohol consumption [7], metabolic syndrome, and obesity as well as from fatty liver disease [6, 8]. Of note, exposure to aflatoxin B1 (AFB1) has also been reported to cause an increased risk for the development of HCC in southern Asia and sub-Saharan Africa [9].

First-line drugs are acting systemically for treating HCC in advanced stages, like sorafenib [10, 11]. This agent is a molecular target specifically for the platelet-derived growth factor receptor-*β* (PDGFR*β*) and vascular endothelial growth factor receptors (VEGFR1/2/3) [12]. Furthermore, its action targets tumor cell proliferation through the inhibition of Raf family kinases (predominantly C-Raf rather than B-Raf) and mainly the RAS/RAF/MEK/ERK pathways, along with angiogenesis pathways via c-KIT, FLT3, and VEGFRs [13]. Because of continuous use, sorafenib has become a drug with an increased high rate of resistance [14]. Among the proteins involved in sorafenib resistance, B-cell lymphoma-2 (Bcl-2), especially the overexpression of Bcl-XL and Mcl-1 are present, which already have been described by [15, 16]. Thus, this review is aimed at describing the influence of the Bcl-2 family proteins in the acquired resistance of HCC.

## 2. Bcl-2 Family: A General Overview

Bcl-2 proteins or Bcl-2 family proteins are typically featured by four conserved Bcl-2 homology (BH) domains, namely, BH1, BH2, BH3, and BH4. These domains consist of eight in total interconnected a-helical fragments. Moreover, an extensively conserved BH domain serves as a crucial foundation for the functionality of Bcl-2 family molecules [17]. Categorizing the Bcl-2 family proteins in mammals has been done based on both homology and function, resulting in three subfamilies including antiapoptotic proteins, for example, Bcl-2 and Bcl-XL; proapoptotic proteins represented by BAX and BAK; and BH3-domain-only proteins like BAD and BID [18].

The Bcl-2 proteins are responsible for modulating a range of processes with its emphasis on the regulation of cell death [19–21]. In the liver, cell death can be induced with a variety of stimuli, for instance, the result of prolonged chronic inflammation [22]. Apoptosis is one of the most well-characterized cell death mechanisms, which regulates tissue homeostasis and surveillance due to its selectivity and, more importantly, the regulation of essential mechanisms in all cells [23–26]. Moreover, Bcl-2 proteins are also involved in the regulation of physiological or pathological mechanisms in relation to immunity and, in some cases, disease development, respectively, by removing damaged cells to reinstate cellular homeostasis [27, 28].

The ability of Bcl-2 proteins to trigger cell death can be initiated through extrinsic and intrinsic pathways. One extrinsic mechanism is the activation of death ligand receptors, located in the cell surface. On the other hand, an intrinsic pathway can be triggered by mitochondrial outer membrane permeabilization (MOMP) [21, 29, 30] leading to cell death driven by bcl-2 antiapoptotic and proapoptotic (prosurvival) proteins. The above is the key to every cell's ability to balance and to maintain appropriate conditions to support cell survival [27]. Thus, the fine affinity among the members of the Bcl-2 proteins drives the interaction associated with pro and antiapoptotic proteins and MOMP in regulating cell fate [31].

A subfamily of Bcl-2 proteins is known for their antiapoptotic characteristics including Bcl-2, Bcl-1, myeloid cell leukemia 1 (Mcl-1), Bcl-w, B-cell lymphoma-extra-large (Bcl-XL), A1, Bcl-x, Burkholderia lethal factor 1 (BLF1/A-1), Bcl-B members, which suppress apoptosis, and are characterized by four domains BH1, BH2, BH3 and BH4 [21, 32, 33]. Furthermore, these proteins maintain the integrity of the mitochondria and avoid the process of cell death [34, 35]. Because of the maintenance of mitochondria membrane integrity, the antiapoptotic characteristics also prevent the release of cytochrome C a protein involved in ATP production and thus, supporting cell viability. [33, 36].

In contrast, the proapoptotic proteins are characterized by their role as inductors of apoptosis, which include Bcl-2-like protein 4 (BAX), Bcl-2 homologous antagonist/killer (BAK) with proeffector function, and Bcl-2 related ovarian killer (BOK) also known as the pore formation proteins [31]. Furthermore, BAK and BAX can have their genes transcribed through the activation of p53 enhancing their levels [37, 38]. The members of the BAX subfamily (BAX, BAK, and BOK) share a structural the sharing combination of the BH1, BH2, and BH3 domains and, thus, are named “multidomain proteins” [39]. In addition, the shared BH3 domain is also termed “BH3-only proapoptotic initiators,” which include Bcl-2-like protein 11 (BIM), Bcl-2-associated death promoter (BAD), BH3 interacting domain death agonist (BID), p53 upregulated modulator of apoptosis (PUMA), Hara-kiri, Bcl-2 interacting protein (HRK), NADPH oxidase activator 1 (Noxa), Bcl-2 interacting killer (BIK), and Bcl-2 modifying factor (BMF) [21, 27, 39–41].

The BH3-only proapoptotic initiating domain is fundamental to mediate the antiapoptotic interaction among these proteins [39] by regulating cytochrome C release from the mitochondria, once its membrane has become disrupted and permeabilized [42]. Members of the BH3-only proteins regulate apoptotic processes through the attraction of BAK or BAX proteins with executing functions, leading to the oligomerization and development of the MOMP [43]. Other studies demonstrated that the proteins termed BNIP3-like protein (BNIP3L) and Bcl-2 19-kDa interacting protein 3 (BNIP3) are additional members of the BH3-only protein family due to a shorter homology sequence of the BH domain [44, 45]. Studies have suggested that the presence of the BH4 domain in some members of the proapoptotic proteins can be an important characteristic to distinguish them between pro and antiapoptotic proteins [42, 46].

A new studied member of the Bcl-2 family is the Bcl-2 L10 protein, also known as Boo, Diva, and Bcl-B [47–49]. In addition, Bcl-2L10 can display different functions which depend on the tumor type where it can act as an oncogenic or tumor suppressor protein [50]. Liu et al. [49], demonstrated that the ongoing methylation of Bcl-2L10 expression was associated with a lower tumor stage, and this was a cancer-specific event. The same group demonstrated particularly in HCC; a tumor suppressor function associated with Bcl-2L10. Others, such as He et al. [51], investigated the role of Bcl-2L10 and BECN1 expression in relation to autophagy in HCC. They revealed that the interaction between Bcl-2L10 and Beclin 1 inhibited the process of autophagy in HCC cells through PI3K/AKT signaling pathway activation resulting in upregulation of mTOR. The overexpression of Bcl-2L10 reduces the activity of HCC cells, in particular when bound to Beclin and PI3KC3 as it decreases their function. This study also analyzed and demonstrated that the level of Bcl-2L10 mRNA in HCC cells and tissue was low. This indicates that a downregulation of Bcl-2 L10 in HCC inhibits autophagy [51].

## 3. The Role of Bcl-2 Family in the Liver: A Physiological View

In a healthy liver and other tissues, apoptosis is controlled by the aforementioned proteins. Those proteins regulate intrinsic and extrinsic pathways, during apoptosis [52]. The absence or deletion of the *bcl-x* gene from hepatocytes results in apoptosis, which can be avoided when deleting specific genes of the BAK and BAX proteins, meaning that the fine balance among the components of the Bcl-2 family control liver apoptosis [52–54]. Moreover, apoptosis of hepatocytes might involve the activation of proteins from the BH3 only family, which is associated with the pathophysiology characteristics of several diseases in the liver [53–56]. The activation of such components in this family e.g., BID, induces apoptosis in healthy liver cells. Whereas, studies using knockout mice, inactivated Mcl-1 and/or Bcl-xL prevented the spontaneous process of apoptosis in hepatocytes [53, 54].

Furthermore, a study utilizing albumin-Cre (Alb-Cre)/Mcl-1^flox/flox^ knockout mice showed that when *Mcl-1* was deleted in the liver epithelium, it resulted in liver damage, apoptosis induction, and the development of HCC, for instance, even though absence of hepatitis evidences [25, 57, 58]. Additionally, the overexpression of the *Mcl-1* minigene in humans leads to apoptosis resistance by promoting liver damage resulting in liver fibrosis [25, 26].

**Figure 1:**
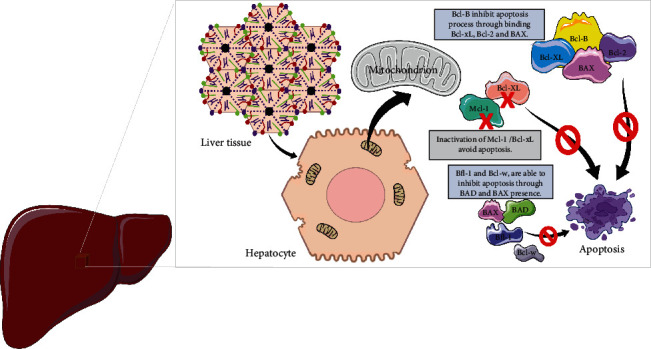
Physiological overview of Bcl-2 family in the liver. Apoptosis in healthy liver is inhibited through binding of Bcl-B to Bcl-xL, Bcl-2, and BAX proteins. Inactivation of Mcl-1 and/or Bcl-xL prevents apoptosis in healthy hepatocytes. Moreover, the overexpression of BAD and/or BAX when together with Bfl-1 and Bcl-w.

On the other hand, overexpression of proteins of the antiapoptotic member including Bfl-1, promotes the survival of hepatocytes [59, 60]. A functional study reported by Luciano and colleagues evidenced that Bcl-B inhibits apoptosis through binding to the proteins Bcl-xL, Bcl-2, and BAX. The Bcl-w protein acts functionally similar to Bcl-2 by promoting cellular survival. Moreover, Bfl-1 and Bcl-w inhibit apoptosis in the presence of BAD or BAX proteins when overexpressed in hepatocytes (Figure 1) [60]. Thus, the apoptotic process is highly regulated, and anti-/proapoptotic proteins are physiologically balanced, which when disrupted, can damage the liver, leading to disease development, including HCC.

## 4. Bcl-2 Family and Hepatocarcinogenesis

The Bcl-2 family has a high relevance in controlling apoptosis through the MOMP pathway. These proteins which belong to the antiapoptotic subfamily, when overexpressed and phosphorylated are linked to cell proliferation, DNA repair, cell cycle, tumorigenesis process, and chemoresistance [6, 61, 62]. In many human tumors, including colon, lung, prostate, neuroblastoma, stomach, lymphoma, breast, and liver cancer, the level of Bcl-2 proteins is increased [63–65].

Studies related to the role of Bcl-2 proteins in HCC showed that healthy hepatocytes did not express proteins of this family. Other studies demonstrated that during cholestasis resulting from toxic bile salts, the expression of Bcl-2 members is associated with apoptosis resistance [66]. Other events in the liver such as the deregulation of lipid metabolism and glucose and the composition of the microbiota can aggravate oncogenesis [67]. Furthermore, the induction of hepatocyte death due to liver damage leads to signalization and activation of Bcl-2 proteins and, as a consequence, the activation of c-Jun N-terminal kinase and caspase [67, 68].

**Figure 2:**
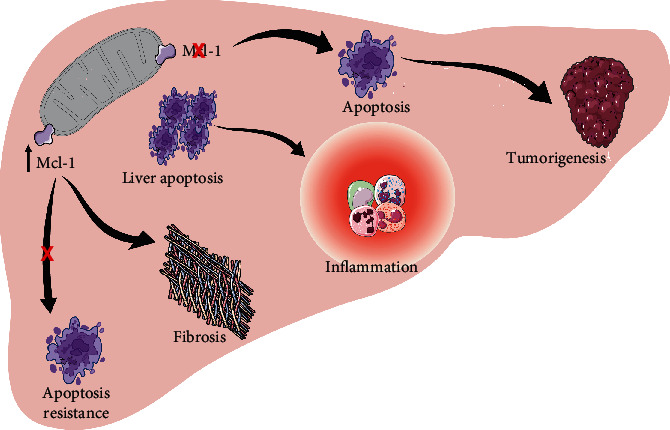
Bcl-2 role in hepatocarcinogenesis. Mcl-1 deficiency results in tumorigenesis due to apoptosis activation and excessive liver death, which trigger the inflammation process. Overexpression of Mcl-1 causes apoptosis resistance and fibrosis formation.

The deficiency of Mcl-1 causes liver tumorigenesis as a result of excessive apoptosis in hepatocytes leading to inflammation and consequently the rise of HCC [69, 70]. Such a deficiency demonstrates an intensification of the phenotype of nonalcoholic steatohepatitis even though it is liver cancer when a NASH model was used [70]. On the other hand, the overexpression of Mcl-1 leads to apoptosis resistance and consequently results in liver damage and the development of hepatic fibrosis (Figure 2) [26, 71, 72], due to the involvement of BOK in hepatocarcinogenesis in mice models when diethylnitrosamine (DEN) was used. Furthermore, their studies also showed that the loss of BOK protein was followed by a decline in HCC cell proliferation both *in vitro* and *in vivo*. BOK-/- mice were protected against apoptosis and inflammation induced by DEN as well as these animals were protected against hepatocarcinogenesis.

The expression level of Bcl-2 in HCC cells has been associated with chemoresistance. Studies lead by Quezada et al. and Whitecross et al. have demonstrated that HCC cells, in which the proapoptotic Bcl-2 protein was overexpressed, showed resistance against the ABT 737 references. ABT 737, a Bcl-2 protein inhibitor, interrupts the binding between Bcl-2/xL and BAK/BAX through competition of the BH3 domain and, consequently, induces apoptosis via the mitochondrial pathway [73, 74]. Ni et al. [75] investigated *in vitro* the ABT 737 sensitivity against the high expression of Bcl-2 in liver cancer cell lines, and they demonstrated that HepG2 and Hep3B cells overexpressed Bcl-2 proteins, which were resistant to ABT 737 and apoptotic pathways. In contrast, Huh-7 and PLC/PRF/5 cells that expressed low levels of Bcl-2 proteins showed sensitivity to this agent, resulting in apoptosis. The same study revealed that apoptosis in those cell lines, with low expression levels of Bcl-2, was utilizing the ROS-JNK pathway.

## 5. Sorafenib and Apoptosis

The first drug with a verified efficacy to treat HCC was sorafenib. Sorafenib is an inhibitor of various multikinase pathways, aiming to inhibit VEGF receptors 1-3, FLT-3, c-Kit, PDGF receptor-band, p38 tyrosine kinases, and one of its main targets, the extracellular kinase receptors and intracellular serine/threonine kinases from Raf/MAPK pathway [76–79]. Furthermore, sorafenib showed to inhibit the Raf/MEK/ERK pathway in PLC/PRF/5 and HepG2 cells, and the phosphorylation of the MEK and ERK proteins [12].

An example of sorafenib and its superior actions was reported in patients diagnosed with an advanced stage of cancer, categorized as stage C according to the Barcelona Clinic Liver Cancer system (BCLC). Its utilization significantly improves the overall survival of individuals undergoing this treatment [80, 81]. A study conducted by Garten et al. [82] observed that sorafenib reduces signaling associated with kinase (ERK) phosphorylation leading to apoptosis in hepatic cell lines such as Hep3B and HUH-7.

Resistance to sorafenib treatment has been demonstrated by Fernando et al. [83], in which. HCC cells presented with a “mesenchymal-like phenotype” and high expression of the CD44. Such high surface expression of CD44 in those mesenchymal-like cells leads to a lower sensitivity to sorafenib and, therefore, less apoptosis [83].

## 6. Sorafenib Resistance and Bcl-2 in HCC

As the first in-line agent to be efficiently approved for HCC treatment [80], sorafenib continues to be the main drug to treat this disease. Unfortunately, it chemoresistance and reduced activity of sorafenib have been reported [6, 84]. A factor involved in sorafenib resistance is hypoxia, which induces the expression of the HIF gene promoting a tumor microenvironment [85–87]. Further factors are oncogenesis, either innate, which occurs at the beginning of the treatment when sorafenib has no action at all, or acquired resistance when the treatment sorafenib becomes ineffective [88–90], as well as miRNA in tissue and bloodstream of patients, and autophagy through the activation of Ras pathways, which are essential factors in this context [91, 92].

Bcl-2 proteins have been associated with acquired sorafenib resistance and HCC proliferation. For example, the overexpression of the Bcl-xL protein has been associated with tumor growth and sorafenib resistance [6, 53, 54, 93]. In addition, high levels of the antiapoptotic protein Mcl-1 were reported in HCC being one of the reasons that cause resistance to apoptosis and against some agents by promoting a malignant phenotype to this disease [94]. Also, the Bcl-2 protein is one of the most important members associated with acquired resistance (not primarily against sorafenib), due to its capacity to inhibit caspase activation through cytochrome c or the entrance of glutathione into the nucleus of HCC cells [95]. Bcl-2 negatively influences CD133^+^ HCC stem cells, which are associated with the activation of the Akt/PKB pathway resulting in HCC cell survival and acquired resistance [96]. A study performed by Chen et al. [97] targeted Mcl-1 proteins, showing that it is a key to reinstall sorafenib sensitivity against recombinant tumor necrosis factor-related apoptosis-inducing ligand (TRAIL). Mcl-1 overexpression in HCC contributes to a malignant phenotype, and the activation of proliferative pathways is, thus, linked to chemotherapeutic resistance [15]. Moreover, Table 1 further summarizes relevant studies associated with Bcl-2 proteins and their important role in sorafenib-acquired resistance.

Sorafenib has been described as an apoptosis inducer in HCC cells, through upregulation of proapoptotic PUMA and BIM, downregulation of antiapoptotic Mcl-1, activation of BAX and BAK, release of cytochrome c, and increase in Caspase-3 function [102]. Sorafenib also contributes to apoptosis in HCC when used in combination with IFN-*α*, due to its suppressive effects and cell cycle arresting, and induction of apoptosis through activation of proteins related to cell cycle arrest such as Bcl-XL, Mcl-1, and Bcl-2 [103]. Furthermore, drugs such as ABT 737, a BH3 inhibitor when associated with sorafenib, are able to induce apoptosis [53, 54].

A study performed by Zhai et al. [104] showed that sorafenib had a low efficacy in downregulating the expression of Bcl-2 and Bcl-xL. In this study, Zhai et al. used arsenic trioxide (ATO), which was able to decrease proteins of the Bcl-2 family and upregulate BAX, demonstrating that this agent induces apoptosis in HCC. In the same study, using ATO synergically with sorafenib was capable of suppressing tumor proliferation resulting in apoptosis of in situ HCC cells. Furthermore, such combination inhibited cyclin D1 disrupting cell cycle arrest in the G0/G1 phase. A recent study by Busche et al. [101] tested HCC cells regarding their sensitivity to a variety of drugs and compared intracellular expression levels of the Bcl-2 members in distinct cell lineages. Their study revealed that Hep3B cells did not express NOXA, one of the Bcl-2 molecules, unlike Huh-7 cells, which present sensitivity to sorafenib combined with TRAIL, suggesting that NOXA may be involved in resistance to such drugs.

Alterations in the cell related to mutations in some genes might be included in the deregulation of Bcl-2 family expression. These mutations are responsible for this imbalance, and it is possible that these are responsible to cause a chemotherapy or radiotherapy-acquired resistance. Low levels of Bcl-xs or BAX expression in HCC cells are associated with P53 mutation, implying that P53 and Bcl-2 family proteins are essential and potentially an alternative target for HCC therapies in the future [95].

## 7. Discussion

Despite the evolution of cancer treatments, HCC still remains one of the deadliest tumors affecting the liver. Its aggressiveness, invasiveness, and extensive dissemination to different organs/tissues result in a poor prognosis for patients [105]. Carcinogenesis is a complex process, causing increased challenges in the diagnosis of cancers due to disease heterogeneity, for example, patient comorbidities, liver dysfunction, and tumor extent [3].

Additionally, treatment options for HCC include traditional tools such as surgery or tissue resection, liver transplantation, radiofrequency ablation (RFA), and percutaneous ethanol injection [106]. Alternatively, the first in-line therapy, approved for advanced HCC in most patients, is sorafenib, which has improved the overall life expectancy of many patients [107]. However, patients usually develop resistance to sorafenib within the first 6 months of therapy, causing a major challenge in the disease prognosis of these patients [108–110]. The development of sorafenib resistance in HCC treatment involves several mechanisms including epigenetic factors, programmed cell death, transportation processes, and the tumor microenvironment [111–116]. In addition, Bcl-2 proteins have shown to be essential in this process due to their regulatory roles and the determination of a cell's live cycle. Those functions are crucial in the development of therapeutic resistance against some chemotherapeutic agents [6, 117].

To overcome these drug resistances, new drugs have been developed, as an important tool known as a second-line treatment against HCC, which include regorafenib [118]. Moreover, this provides HCC patients with an alternative treatment option [6]. Finn et al. discussed regorafenib and cabozantinib and their action to be used as second-in-line drugs for patients who progress with sorafenib during the treatment of HCC. Regorafenib, however, is a multitargeted tyrosine kinase inhibitor similar to sorafenib. Lenvatinib, another emerging therapeutic drug, shows promising advantages in reducing drug resistance [119].

Al-Salama and colleagues described that lenvatinib targets VEGFR1-3, FGF receptors 1-4, platelet-derived growth factor receptor-*α*, RET, and cKIT overcoming the development of drug resistance during the treatment. In addition, ramucirumab (a recombinant monoclonal antibody) is also an alternative therapeutic, and unlike any other second-in-line drug, it targets VEGFR2 by blocking the interaction with its ligand and their downstream signaling exerting an antitumor effect [120]. The above drugs provide an alternative to the first in-line sorafenib, and its resistance, by improving patients' survival and overall life expectancy. However, a disadvantage and limiting factor for most patients is the high treatment costs associated with such newer drugs.

Nevertheless, sorafenib resistance can be reverted using some inhibitors and an important alternative to overcome the aforementioned obstacles. Regarding Bcl-2 proteins and their influence in front of sorafenib/regorafenib efficacy, some drugs as BH3 mimetics have been interesting to be used as a single or associated therapy with sorafenib [6]. The BH3 mimetics have been in some trials, and some others, such as Bcl-xL inhibitors, have been as well. Venetoclax (ABT199) was approved to be used for leukemia for example [121–123].

However, the combination of sorafenib/venetoclax has not been an interesting therapeutic alternative; although the ratio Bcl-2/Mcl-1 can be identified as a marker for sorafenib activity, it is necessary for the simultaneous inhibition of Bcl-xL, which potentiates the sorafenib effect as discussed by Tutusaus et al. in their study. One of the reasons for the lack of efficacy when performing such a combination is that HCC behaves in a “BCL-xL-dependent tumor,” according to Tutusaus et al. In fact, testing such combination might be of most important because it may overcome sorafenib resistance. Navitoclax (ABT-263) was also used in a clinical trial to revert sorafenib resistance; however, it resulted in some cytotoxicity, although manageable, for patients and presented in thrombocytopenia [124].

In summary, sorafenib has been the first-in-line drug to treat HCC in an advanced stage. Sorafenib is a molecular drug to treat this disease; however, the drug resistance has become an ongoing problem. The acquired drug resistance is due to different factors but is mostly associated with proteins of the Bcl-2 family. Most of them are related to overexpression, resulting in inhibition of cellular apoptosis, which leads to resistance. Therefore, targeting the overexpression of proteins of the Bcl-2 members in HCC might be an effective treatment. It seems still a challenging and complex process to overcome sorafenib resistance and the inhibition of Bcl-2 proteins; however, new clinical trial studies are needed as potential alternatives for sorafenib therapy especially for patients with an advanced stage HCC.

## Figures and Tables

**Table 1:** Key studies on Bcl-2 and HCC sorafenib resistance.

**Focus**	**Methods**	**Main results**	**References**
Presents about the effects of iron deficiency on sorafenib resistance.	Use of hepatoma cell lines (Huh7 and Hep3B) treated with sorafenib and PX478.	-Offers a viewpoint about the upregulation of antiapoptotic BCL-2 protein in the presence of iron deficiency, followed by downregulation of expressions in apoptotic proteins, such as BAX, Caspase-3, and Caspase-9; -Iron increases hypoxia-inducible factor 1 alpha (HIF-1*α*), and BCL-2 levels; -Suppression of HIF-1*α* reduced the iron deficiency-induced BCL-2 expression and the sorafenib resistance.	[98]

Discuss the occurrence of BCL-2 and sorafenib resistance in HCC; and the advantages of the BH3-mimetics associated with sorafenib.	Performing with Human liver tumor cell lines (Hep3B, PLC, and HepG2) treated with sorafenib. Also, male Swiss nude mice were used, where HepG2 or BCLC9 cells were injected subcutaneously.	-Introduces about hepatoma cells' resistance to sorafenib (HepG2R and Hep3BR); -Sensibilization of cancer cells drug-resistant to apoptosis through BH3-mimetics; -Mouse xenograft models from patient-derived BCLC9 cells responded to sorafenib, and an association linked to changes in the BCL-2 mRNA pattern was observed.	[6]

Testing the innovative activity of amygdalin (Amy) and/or sorafenib (Sor) against HCC using the HepG2 cell line.	The HepG2 and normal lung WI-38 human cell lines. Amygdalin and sorafenib tosylate were used in HepG2 and WI-38 cells.	-Argument about cytotoxic selectivity against HepG2 cells compared to normal cells treated with sorafenib and amygdalin was performed. -Conversely, WI-38 cells exhibited greater sensitivity to Sorafenib toxicity. -Interaction of both drugs was linked to cell cycle arrest at the S and G2/M stages, increased apoptosis, and potential necroptosis.	[99]

Investigate the function of BAD in a panel of HCC cell lines.	Samples of HCC and nontumor tissue were obtained from surgical resections from the Amiens University Hospital, France.	-Evidence of the expression of BCL2 proteins and decrease of BAD expression, (a proapoptotic BH3-only member of the BCL2 family), in HCC, examined the relevance of this decrease and analyzed its therapeutic implications.	[100]

Investigation using HCC cell lines and tissues from patients, treated with sorafenib combined with apoptosis-inducing or sensitizing agents, such as TRAIL or BH3-mimetic ABT-737.	Cell lines: Huh7, Hep3B, HLE, HepG2, and HLF, and primary human hepatocytes (PHHs) or human HCC tissues were isolated from liver tissues from partial hepatectomy. Treatment performed with Sorafenib, TRAIL, and ABT-737	-Suggestions regarding the BH3-only protein expression may determine the response of HCC treatment to different sorafenib-based drug combinations.	[101]

Analyzing the effects of sorafenib on autocrine proliferation and survival of different human HCC cell lines.	Human cell lines (Hep3B, HepG2, SK-Hep1, and PLC/PRF/5) were used and treated with sorafenib tosylate. Human recombinant TGF-b1 and TNF were used after sorafenib treatment.	-Discussion about the counteraction of sorafenib (*in vitro)* with autocrine growth of different tumor cells (Hep3B, HepG2, PLC- PRF-5, SK-Hep1). -Arrests in S/G2/M cell cycle phases coincide with cyclin D1 downregulation. -Sorafenib's activity seems to occur through cell death induction, which correlates with caspase activation, increase in the percentage of hypodiploid cells, activation of BAX and BAK, and cytochrome c release from mitochondria to cytosol. -A rise in mRNA and protein levels of the proapoptotic “BH3-domain only” PUMA and BIM, reduced protein levels of antiapoptotic MCL1 and surviving. PUMA targeting knock-down, by using specific siRNAs, inhibited sorafenib-induced apoptotic features.	[102]

*In vivo* evidence that overexpression of Bcl-xL is directly associated with the growth of solid tumors. ABT-737, Bcl-xL, an inhibitor, but not Mcl-1, could control HCC progression, especially when combined with sorafenib.	Bcl-xL knockout mice (Bcl-x^flox/flox^Alb-Cre [albumin/cre recombinase]) and Mcl-1 knockout mice (mcl-1^flox/flox^ Alb-Cre), Balb/c nude mice (CAnN.Cg-Foxn1^nu^/CrlCrlj) were used.	-Argued about sorafenib downregulated Mcl-1 expression in tumor cells and decreased Mcl-1 upregulation induced by ABT-737 (BH3 mimetic). -Sorafenib, in combination with ABT-737, induced apoptosis in hepatoma cells. -This combination suppresses xenograft tumors rather than sorafenib alone. Bcl-xL inactivation by the combination was described as safe and effective for anti-HCC therapy in preclinical models.	[53, 54]

## Data Availability

The authors have nothing to report.

## Nomenclature

BAD: Bcl-2-associated death promoter
BAK: Bcl-2 homologous antagonist/killer
BAX: Bcl-2-like protein 4
BCLC: Barcelona Clinic Liver Cancer
Bcl-XL: B-cell lymphoma-extra-large
BID: BH3 interacting domain death agonist
BIK: Bcl-2 interacting killer
BIM: Bcl-2-like protein 11
BLF1/A-1: Burkholderia lethal factor 1
BMF: Bcl-2 modifying factor
BNIP3: Bcl-2 19-kDa interacting protein 3
BNIP3L: BNIP3 like protein
BOK: Bcl-2-related ovarian killer
DEN: diethylnitrosamine
HGF: hepatocyte growth factor
HIF-2*α*: hypoxia-inducible factors 2*α*
HRK: Bcl-2 interacting protein
Mcl-1: myeloid cell leukemia 1
MOMP: out membrane permeabilization
NASH: nonalcoholic steatohepatitis
Noxa: NADPH oxidase activator 1
PUMA: p53 upregulated modulator of apoptosis
TRAIL: recombinant tumor necrosis factor-related apoptosis-inducing ligand
